# Prevalence of atrial fibrillation dependent on coronary artery status: Insights from the LIFE‐Heart Study

**DOI:** 10.1002/clc.23490

**Published:** 2020-10-27

**Authors:** Jelena Kornej, Sylvia Henger, Timm Seewöster, Andrej Teren, Ralph Burkhardt, Holger Thiele, Joachim Thiery, Markus Scholz

**Affiliations:** ^1^ National Heart, Lung, and Blood Institute's Framingham Heart Study Framingham Massachusetts USA; ^2^ Sections of Cardiovascular Medicine and Preventive Medicine, Boston Medical Center Boston University School of Medicine Boston Massachusetts USA; ^3^ LIFE – Leipzig Research Center of Civilization Diseases Leipzig University Leipzig Germany; ^4^ Institute for Medical Informatics, Statistics and Epidemiology Leipzig University Leipzig Germany; ^5^ Department of Electrophysiology Heart Center at University of Leipzig Leipzig Germany; ^6^ Institute of Clinical Chemistry and Laboratory Medicine University Hospital Regensburg Regensburg Germany; ^7^ Department of Internal Medicine/Cardiology Heart Center at University of Leipzig Leipzig Germany

**Keywords:** atrial fibrillation, CAD extent, CAD origin, coronary artery disease, coronary artery sclerosis, prevalence

## Abstract

**Background:**

Coronary artery disease (CAD) is a significant risk factor for atrial fibrillation (AF). Experimental studies demonstrated that atrial ischemia induced by right coronary artery (RCA) stenosis promote AF triggers and development of electro‐anatomical substrate for AF.

**Aim:**

To analyze the association between AF prevalence and coronary arteries status in the LIFE‐Heart Study.

**Methods:**

This analysis included patients with available coronary catheterization data recruited between 2006 and 2014. Patients with acute myocardial infarction were excluded. CAD was defined as stenosis ≥75%, while coronary artery sclerosis (CAS) was defined as non‐critical plaque(s) <75%.

**Results:**

In total, 3.458 patients (median age 63 years, 34% women) were included into analysis. AF was diagnosed in 238 (6.7%) patients. There were 681 (19.7%) patients with CAS and 1.411 (40.8%) with CAD (27.5% with single, 32.4% with double, and 40.1% with triple vessel CAD). In multivariable analysis, there was a significant association between prevalent AF and coronary artery status (OR 0.64, 95% CI 0.53‐0.78, *P*_trend_ < .001). Similarly, AF risk was lower in patients with higher CAD extent (OR 0.54, 95%CI 0.35‐0.83, *P*_trend_ = .005). Compared to single vessel CAD, the risk of AF was lower in double (OR 0.42, 95%CI 0.19‐0.95, *P* = .037) and triple CAD (OR 0.31, 95%CI 0.13‐0.71, *P* = .006). Finally, no association was found between AF prevalence and CAD origin among patients with single vessel CAD.

**Conclusion:**

In the LIFE‐Heart Study, CAS but not CAD was associated with increased risk of AF.

**Abbreviations**
ACE/ARB: angiotensin‐converting‐enzyme/angiotensin receptor blockers
AF: atrial fibrillation
CAD: coronary artery disease
CAS: coronary artery sclerosis
eGFR: estimated glomerular filtration rate
LA: left atrial/‐um
LV‐EF: left ventricular ejection fraction
SNA: sinus node artery

## 1 INTRODUCTION

Coronary artery disease (CAD) is a very common cardiovascular disease, while atrial fibrillation (AF) is the most prevalent sustained cardiac arrhythmia in adults.^1^ Both diseases share common risk factors including hypertension, diabetes mellitus, sleep apnea, obesity, and smoking.^2^ There are several animal experimental studies demonstrating the relationship between chronic atrial ischemia and AF substrate.^3^, ^4^ In particular, right atrial ischemia induced by right coronary artery (RCA) stenosis has been shown to promote AF triggers and a substrate for AF maintenance.^4^ Although *atrial* myocardial infarction (MI) is considered as unusual and is often undetected, the largest series of autopsy‐assessed *atrial* infarctions performed in the early 1940s indicated an incidence of 17%.^5^ Acute atrial ischemia creates a substrate for AF maintenance within several hours^6^, ^7^ leading to decreased conduction velocity and increased conduction heterogeneity caused by hypoxia and atrial effective refractory period (ERP) shortening.^3^, ^8^

There are only few small clinical studies analyzing associations between AF recurrences and CAD or coronary artery sclerosis (CAS), and the results are inconsistent. One study analyzed the impact of the origin of sinus node artery (SNA) on AF recurrence after pulmonary vein isolation in patients with paroxysmal AF and found that left SNA group (SNA originating from the left circumflex artery) is more frequent in patients with paroxysmal AF.^9^ Another study with 125 patients investigated an impact of CAS on the efficacy of AF radiofrequency catheter ablation and found that CAS was not useful to predict rhythm outcomes thereafter.^10^ Analyzing the impact of stable CAD on rhythm outcomes in a clinical cohort of over 1.300 patients undergoing AF catheter ablation, there was no association between CAD presence, origin/extent and AF recurrences.^11^ However, the study included only ~12% of AF patients with known CAD.

## 2 AIM

The aim of the study was to analyze association between AF prevalence and coronary arteries status in patients undergoing invasive coronary diagnostic within the framework of the LIFE‐Heart Study. We hypothesized that CAD presence and extent are associated with higher risk of AF. Furthermore, we analyzed whether CAD origin is associated with prevalent AF.

## 3 METHODS

### 3.1 Study population

**FIGURE 1:**
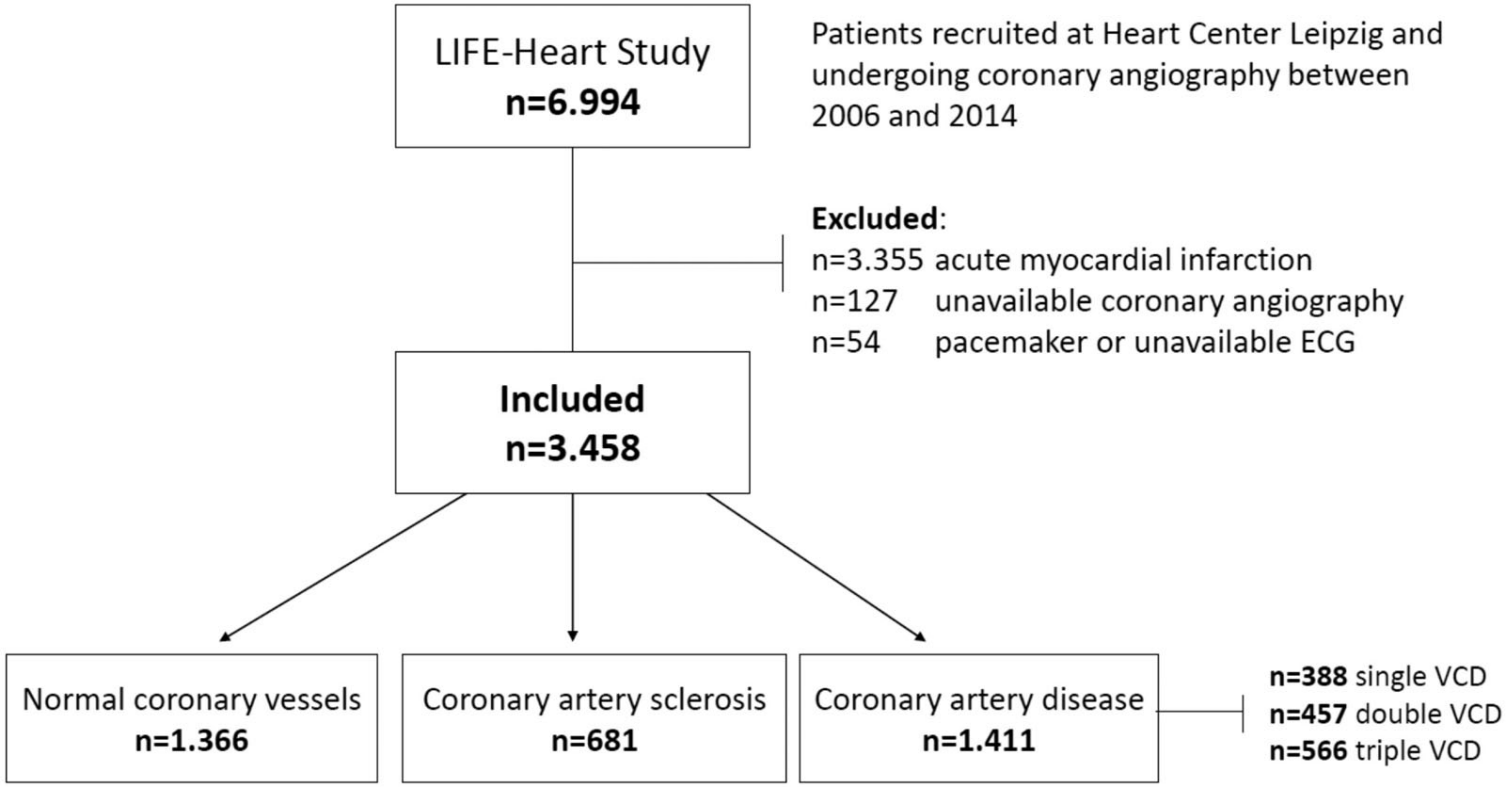
Study flowchart

The LIFE‐Heart Study is a mono‐centric observational study of patients with confirmed or suspected CAD. Patients were recruited between 2006 and 2014. In total, LIFE‐Heart Study included 6.994 patients. Study details are presented elsewhere.^12^ Patients with acute MI, unavailable coronary angiography data or ECGs were excluded from the present analysis. The final analyzed sample consisted of 3.458 patients with available clinical, echocardiographic, laboratory data, and known coronary status (Figure 1).

The study was approved by the local Ethic Committee, and all patients provided written informed consent for participation. All methods were performed in accordance with the relevant guidelines and regulations.

### 3.2 Definitions

CAD was defined as clinically relevant stenosis with ≥75% luminal reduction, while CAS was defined as a non‐critical plaque with <75% luminal reduction. Furthermore, dependent on obstructed vessels number, CAD extent was defined as single vessel disease—if one coronary vessel was obstructed, as double vessel disease—if two coronary vessels or left main stem (LMS) were obstructed, or as triple vessel disease—if all three coronary vessels (RCA, LAD, CX) or RCA with LMS were obstructed. Finally, CAD origin was defined accordingly to the origin of the obstructed vessel (eg, RCA, LAD, and CX). Unobstructed (normal) coronary arteries were vessels without visible luminal irregularities. AF was defined, if irregular atrial rhythm with f‐waves was documented in resting ECGs prior to coronary artery catheterization.

### 3.3 Laboratory measurements

Blood was drawn prior to invasive coronary diagnostic. All samples were processed in a highly standardized manner as previously described.^13^ Laboratory measurements of creatinine serum concentrations were performed on the same day at the Institute of Laboratory Medicine, University Hospital Leipzig (accredited by ISO 15189 and 17025) according to the Quality Standards for Medical Laboratories of the German Chamber of Physicians (RiLiBÄK) using assays from Roche Diagnostics on Cobas 6000 or 8000 (Roche Diagnostics) clinical chemistry analyzers. Estimated glomerular filtration rate (eGFR) was analyzed using the Chronic Kidney Disease Epidemiology Collaboration equation.^14^

### 3.4 Statistical analysis

Data are presented as median (interquartile range) and as percentages. We performed unadjusted univariable analysis to analyze association of AF with (a). coronary artery status (normal [=unobstructed] coronary arteries, CAS, and CAD) in the whole study population; (b). CAD extent (single, double, and triple coronary vessel disease) in patients with CAD; and (c). CAD origin (RCA, LAD, CX) in patients with single coronary vessel disease. Also, we performed three multivariable analyses applying generalized models and trend test with proportional odds using logistic regression of vessel status (Model 1—adjusted for age and sex, Model 2—Model 1 and further adjustement for BMI, hypertension, diabetes mellitus, current smoking, eGFR, LA diameter, EF, usage of ACE/AR blockers, beta blockers, and statins, and Model 3—Model 2 and further adjustement for CRP and IL‐6). Medication use was tested accordingly to coronary artery status using linear‐by‐linear association with *χ* ^2^ test.

A *P* value <.05 was considered statistically significant. Analyses were performed with SPSS statistical software version 26 (SPSS Inc., Chicago, IL) and the statistical software package R.

## 4 RESULTS

### 4.1 Clinical characteristics of study population

There were 3.458 patients (median age 63 years [interquartile range 55‐71], 34% women) included into analysis. Baseline clinical characteristics of the study cohort are summarized in Table 1. AF was diagnosed in 238 (6.9%) patients. According to the coronary artery status, 1.366 (39.5%) patients had unobstructed coronary vessels, CAS/non‐critical CAD was found in 681 (19.7%) patients, while 1.411 (40.8%) patients had CAD (among them 388 (27.5%) had single vessel CAD, 457 (32.4%) double, and 566 (40.1%) triple vessel CAD). Compared to the non‐AF group, patients with AF were significantly older, less frequently females, and had more often unfavorable cardiovascular profile (higher BMI, lower eGFR, more often hypertension, and diabetes mellitus). Echocardiographic LA diameter was significantly higher and LV‐EF was lower among patients with AF. Finally, CRP and IL‐6 were significantly higher in patients with AF (Table 1).

**TABLE 1:** Baseline characteristics of the study population accordingly to AF presence

	Total study cohort n = 3.458	AF n = 238	Non‐AF n = 3.220	*P* value
Age (years)	63 (55‐71)	70 (61‐76)	63 (55‐71)	<.001
Females (%)	34.3	27.7	34.8	.027
Body mass index (kg/m^2^)	29.0 (26.1‐32.5)	30.0 (27.3‐33.6)	28.9 (26.0‐32.3)	<.001
Hypertension (%)	82.1	90.8	81.4	<.001
Diabetes mellitus (%)	31.4	48.7	30.1	<.001
Current smoker (%)	19.1	14.3	19.5	<.001
eGFR (mL/min/1.73 m^2^)	85 (72‐96)	78 (64‐89)	86 (72‐96)	<.001
Coronary artery status				.002
Unobstructed coronary vessels (%)	39.5	39.9	39.5	
CAS/non‐critical CAD (%)	19.7	32.4	18.8	
CAD≥75% (%)	40.8	27.7	41.8	
CAD extent^a^				.197
Single	27.5	34.8	27.1	
Double	32.4	30.3	32.5	
Triple	40.1	34.8	40.4	
CAD origin^b^				.307
RCA	61.5	54.5	61.9	
LAD	74.9	65.2	75.4	
CX	60.9	66.7	60.6	
Medication				
ACE/AR blockers (%)	71.1	81.1	70.4	<.001
Beta blockers (%)	60.4	73.9	59.4	<.001
Lipids lowering medication (%)	40.6	38.2	40.8	.436
Echocardiographic data				
LA diameter (mm)	39 (36‐43)	48 (44‐53)	39 (35‐43)	<.001
LV‐EF (%)	61 (55‐65)	54 (46‐63)	61 (56‐66)	<.001
C‐reactive protein (mg/L^c^)	2.5 (1.2‐5.3)	3.0 (1.7‐9.4)	2.4 (1.1‐5.2)	.024
Interleukin‐6^d^	3.1 (1.7‐6.1)	5.0 (2.6‐10.1)	3.1 (1.7‐5.9)	<.001

*Note:* Data presented as mean (interquartile range) or %.

Abbreviations: ACE/ARB, ACE‐inhibitors/angiotensin receptor blockers; AF, atrial fibrillation; CAD, coronary artery disease; CAS, coronary artery sclerosis; eGFR, estimated glomerular filtration rate; LA, left atrial; LV‐EF‐ left ventricular ejection fraction.

^a^ In patients with coronary artery disease (n = 1.411).

^b^ In patients with single vessel disease (n = 388).

^c^ Data available in n = 2.173.

^d^ Data available in n = 2.188.

Differences in baseline characteristics between the groups of different coronary arteries status are summarized in Table 2. Compared to patients with unobstructed coronary vessels, patients with CAS and CAD were significantly older, were more frequently men, had more often hypertension, diabetes as well as antihypertensive and lipid lowering medication (all *P* < .001). Among inflammatory markers, only IL‐6 was significantly higher in patients with CAD.

**TABLE 2:** Baseline characteristics of the study population accordingly to coronary artery status

	Unobstructed coronary vessels n = 1.366	Coronary artery sclerosis or non‐critical stenosis n = 681	Coronary artery disease n = 1.411	*P* value
Age (years)	59 (52‐68)	65 (57‐72)	66 (58‐73)	<.001
Females (%)	49.2	30.7	21.6	<.001
Body mass index (kg/m^2^)	29.0 (25.7‐32.6)	29.6 (26.7‐33.1)	28.7 (26.1‐32.0)	.001
Hypertension (%)	76.6	85.3	85.8	<.001
Diabetes mellitus (%)	22.7	34.0	39.9	<.001
Current smoker (%)	16.8	20.0	20.9	.130
eGFR (mL/min/1.73 m^2^)	89 (76‐98)	83 (70‐95)	82 (68‐93)	<.001
Atrial fibrillation	7.0	11.3	4.7	.002
Medication				
ACE/AR blockers (%)	64.6	74.7	75.6	<.001
Beta blockers (%)	55.6	60.5	65.1	<.001
Lipids lowering medication (%)	29.4	40.2	51.7	<.001
Echocardiographic data				
LA diameter (mm)	38 (35‐42)	40 (36‐45)	40 (36‐44)	<.001
LV‐EF (%)	61 (56‐66)	61 (56‐65)	60 (54‐65)	<.001
C‐reactive protein (mg/L^a^)	2.4 (1.1‐5.1)	2.4 (1.2‐4.8)	2.5 (1.2‐5.6)	.902
Interleukin‐6^b^	2.7 (1.7‐4.7)	2.8 (1.6‐4.9)	3.4 (1.8‐6.7)	<.001

Abbreviations: ACE/ARB, ACE‐inhibitors/angiotensin receptor blockers; eGFR, estimated glomerular filtration rate; LA, left atrial; LV‐EF, left ventricular ejection fraction.

^a^ Data available in n = 2.173 individuals.

^b^ Data available in n = 2.188 individuals.

### 4.2 Association between coronary artery status and AF

Table 3 presents different models analyzing association between prevalent AF and coronary artery status. In multivariate analysis of the full model, there was a significant association between prevalent AF and coronary artery status (OR 0.64, 95% CI 0.53‐0.78, *P*_trend_ < .001). However, there was no significant difference of AF risk between patients with unobstructed coronary vessels and CAS or CAD (OR 1.34, 95% CI 0.59‐3.04 and OR 0.45, 95%CI 0.20‐1.02, respectively). Compared to patients with CAS, patients with CAD had lower AF risk (OR 0.33, 95% CI 0.21‐0.52, *P* < .001).

**TABLE 3:** Association between AF and coronary artery status, CAD extent and origin

	Unadjusted		Model 1		Model 2		Model 3	
	OR (95% CI)	*P* value	OR (95% CI)	*P* value	OR (95% CI)	*P* value	OR (95% CI)	*P* value
Association between *coronary artery status* (unobstructed coronary arteries, coronary artery sclerosis, coronary artery disease) and AF in the whole study cohort (n = 3.458)								
Trend test^a^	0.85 (0.77; 0.94)	.002	0.72 (0.64; 0.80)	<.001	0.70 (0.61; 0.81)	<.001	0.64 (0.53; 0.78)	<.001
Normal vs CAS^b^	1.71 (1.24; 2.34)	.001	1.16 (0.84; 1.62)	.366	1.14 (0.76; 1.71)	.516	1.34 (0.59; 3.04)	.479
Normal vs CAD^b^	0.66 (0.48; 0.91)	.011	0.39 (0.28; 0.56)	<.001	0.36 (0.24; 0.56)	<.001	0.45 (0.20‐1.02)	.057
CAS vs CAD^b^	0.39 (0.27; 0.54)	<.001	0.34 (0.24; 0.48)	<.001	0.32 (0.20; 0.50)	<.001	0.33 (0.21; 0.52)	<.001
Association between *coronary artery disease extent* (single, double, triple vessel disease) and AF in the sub‐cohort with CAD (n = 1.411)								
Trend test^a^	0.82 (0.61; 1.11)	.198	0.78 (0.57; 1.06)	.109	0.56 (0.37; 0.85)	.007	0.54 (0.35; 0.83)	.005
Single vs double^b^	0.73 (0.39; 1.34)	.308	0.65 (0.35; 1.21)	.177	0.42 (0.19; 0.94)	.036	0.42 (0.19; 0.95)	.037
Single vs triple^b^	0.67 (0,37; 1.22)	.189	0.60 (0.33; 1.10)	.099	0.33 (0.15; 0.74)	.007	0.31 (0.13; 0.71)	.006
Double vs triple^b^	0.93 (0.50; 1.71)	.804	0.93 (0.50; 1.72)	.805	0.78 (0.33; 1.89)	.585	0.73 (0.30; 1.79)	.491
Association between *coronary artery disease origin* (RCA, LAD, CX stenosis) and AF in the sub‐cohort with a single CAD (n = 388)								
Trend test^a^	1.39 (0.74; 2.60)	.309	1.37 (0.72; 2.62)	.334	1.73 (0.79; 3.80)	.169	1.62 (0.71; 3.69)	.248
RCA vs LAD^b^	0.57 (0.20; 1.62)	.293	0.54 (0.19; 1.56)	.254	0.96 (0.23; 4.00)	.950	0.75 (0.17; 3.25)	.698
RCA vs CX^b^	1.84 (0.63; 5.32)	.263	1.83 (0.61; 5.49)	.279	2.79 (0.63; 12.42)	.177	2.38 (0.51; 11.06)	.271
LAD vs CX^b^	0.31 (0.11; 0.86)	.025	0.29 (0.10; 0.84)	.023	0.34 (0.09; 1.30)	.114	0.32 (0.08; 1.22)	.095

*Note:* Model 1—adjusted for age and sex; Model 2—Model 1 + BMI, hypertension, diabetes, current smoking, eGFR, LA diameter, ejection fraction, medication (ACE/ARB, beta blockers, and statins); Model 3—Model 2 + CRP and IL‐6.

Abbreviations: ACE/ARB, ACE‐inhibitors/angiotensin receptor blockers; AF, atrial fibrillation; CAD, coronary artery disease; RCA, right coronary artery.

^a^ Trend tested with proportional odds using logistic regression of vessel status.

^b^ The first category treated as reference.

Analyzing association between CAD extent (single, double, triple vessel disease) and AF risk in the subgroup with relevant CAD, we found lower AF risk in patients with advanced CAD extent (OR 0.54, 95% CI 0.35‐0.83, *P* = .005). Compared to single vessel CAD, the risk of AF was lower in double (OR 0.42, 95%CI 0.19‐0.95, *P* = .037) and triple CAD (OR 0.31, 95% CI 0.13‐0.71, *P* = .006). No difference in AF prevalence was found between patients with double and triple CAD.

Finally, no significant association was found between CAD origin and AF risk in the subgroup of patients with single vessel disease.

### 4.3 Impact of medication

To investigate whether our findings could be driven by differences in medication with pleiotropic(lipid lowering drugs and ACE/ARBs) or negative chronotropic effect (beta blockers), we analyzed medication in the whole cohort as well as in the subgroup of patients with CAD and single vessel CAD (Table 4). We found significant differences of medication usage between groups of coronary artery status. Although the proportion of patients without any medication was expectedly higher in patients with normal (unobstructed) coronary vessels (54%), over 1/5 of these patients took all three drugs. In contrast, while usage of all three medications was the highest in patients with critical CAD (59%), almost 1/3 did not take any of these drugs. Analyzing differences in medication in the subgroup with CAD, all three medications were used by 27%, 36%, and 43% patients with single, double, and triple vessel CAD, respectively. Finally, no difference in medication was found among the groups of CAD origin in the subgroup of patients with single vessel CAD.

**TABLE 4:** Medication use accordingly to coronary artery status

Medication: Beta blockers ACE/ARB Lipids lowering medication	None	1 of 3	2 of 3	3 of 3	*P* value
Coronary artery status in the whole cohort (n = 3.458)					<0.001^a^
Unobstructed coronary vessels	54.3	39.5	38.0	22.8	
CAS/non‐critical CAD	14.2	20.1	19.0	18.1	
CAD≥75%	31.5	40.4	43.1	59.1	
CAD extent in a subgroup with CAD (n = 1.411)					<0.001^a^
Single vessel CAD	13.1	27.6	34.5	27.4	
Double vessel CAD	9.4	22.3	32.4	35.9	
Triple vessel CAD	8.1	21.0	28.3	42.6	
CAD origin in a subgroup with one vessel CAD (n = 388)					0.687^a^
RCA	10.5	27.6	36.2	25.7	
LAD	15.2	25.5	34.8	24.5	
CX	11.6	30.4	31.9	26.1	

*Note:* Data presented in %.

Abbreviations: ACE/ARB, ACE‐inhibitors/angiotensin receptor blockers; AF, atrial fibrillation; CAD, coronary artery disease; CAS, coronary artery sclerosis; eGFR, estimated glomerular filtration rate; LA, left atrial; LV‐EF‐ left ventricular ejection fraction.

^a^ Trend tested as linear‐by‐linear association with *χ* ^2^ test.

## 5 DISCUSSION

### 5.1 Main findings

In current study we analyzed an association between AF prevalence and coronary arteries status in a large clinical cohort undergoing coronary artery catheterization. We found that patients with CAS had more often AF compared to patients with unobstructed coronary vessels or CAD. Also, compared to patients with single vessel CAD, the risk for AF was lower in those with double and triple CAD. Finally, there was no association between AF and CAD origin among patients with single vessel CAD.

### 5.2 AF and coronary artery disease

CAD is considered as a relevant risk factor for AF.^15^ However, *atrial* MI is less investigated because of difficulties in detection. According to the experimental findings, acute atrial ischemia creates a substrate for AF maintenance within several hours.^6^ There is an evidence that RCA occlusion promotes AF triggers and substrate formation facilitating AF initiation and maintenance.^4^ The leading mechanisms for underlying arrhythmogenesis supposed to be a triggered activity with consequent generation of spontaneous atrial firing and border zone reentry that promotes AF‐maintaining reentrant sources. While some groups observed inhomogeneous refractory periods, decreased conduction velocity and increased conduction heterogeneity caused by hypoxia in rabbits,^8^ others demonstrated that proximal RCA occlusion in dogs causes atrial ERP shortening within several hours.^3^

However, the impact of CAD on AF treatment and outcomes is understudied in clinical setting. Recently, Zhang et al analyzed the impact of the origin of SNA on AF recurrence after pulmonary vein isolation in patients with paroxysmal AF.^9^ The SNA is a major artery of the atrial coronary circulation. Coronary angiography and postmortem studies found that the SNA originates from the RCA in the majority (51%‐61%) of the patients, and in others from the LCX (35%‐42%).^16^ Zhang et al reported a higher percentage (~56%) of the SNA arising from the LCX in patients with *paroxysmal* AF.^9^ The authors found that beside LA size (HR 1.45, *P* < .001), the left SNA (HR 6.22, *P* = .002) remained an independent predictor for AF recurrences. However, these results could be different in patients with persistent AF. Also, because of relatively small sample size (<100 patients), the results require validation in larger external cohorts.

Another clinical study analyzed an impact of the presence and extent of stable CAD on arrhythmia recurrences during 12 months follow‐up after AF catheter ablation.^11^ Stable CAD was not associated with occurrence of arrhythmia recurrences. Furthermore, among patients with CAD, neither the origin nor the extent were related to rhythm outcomes after AF catheter ablation. However, the main limitation of the study was its retrospective design and only ~12% of AF cases with stable CAD. Therefore, these results may not reflect the true CAD effect on arrhythmia recurrences after AF ablation.

The present analysis with >3.400 patients does not support our initial hypothesis and contradicts previous results.^4^, ^9^ We found that the risk for AF was 67% lower in patients with CAD compared to CAS. In line with this observation, we found that the risk for AF was lower in patients with more advanced CAD (double and triple vessel CAD) compared to those with single vessel CAD. A possible explanation is the action of ACE/ARB and statins treatment known for their pleiotropic effects in AF patients.^17^ In our study, the prevalence of ACE/ARBs, beta blockers, and statins use was significantly higher in patients with CAD. As expected, the proportion of the triple drug combination was higher in patients with advanced CAD. We speculate that pleiotropic effects of these drugs might at least partly explain significantly lower AF incidence in CAD patients. However, adjustment for drug usage (in model 2) did not essentially change effect sizes. Another explanation could be sub‐clinical inflammation, although there was again not relevant change in the results after adjustment for the inflammatory markers CRP and IL‐6 (in model 3). We conclude that the observed differences in AF risk could not be explained by differences in medication or inflammatory status. Finally, we suspect that patients with CAS underwent coronary angiography very likely because of AF paroxysms, which share similar clinical symptoms with CAD such as chest discomfort, dyspnea, and anxiety. Therefore, our observation could be a result of selection bias of patients with cardiac discomfort or unspecific signs of cardiovascular disease requiring invasive diagnostics by coronary angiography.

### 5.3 AF and coronary artery sclerosis

There are considerably less data regarding the impact of CAS on AF prevalence. One clinical study investigated the role of CAS in patients undergoing AF catheter ablation.^10^ The authors found that non‐significant CAD (vessel obstruction <50%) was similar in patients with and without AF recurrences (45% and 47%, respectively). In our study, we found higher AF prevalence in patients with CAS compared to CAD. Also, in multivariable analysis, patients with CAD had significantly lower risk for AF than those with CAS. Although patients with CAS had almost 2‐fold risk for AF compared to normal vessels in univariable analysis, after multivariable adjustments this association was no longer significant. As already discussed above, pleiotropic effects of ACE/AR blockers and statins may partly explain these findings.^17^

### 5.4 Strengths and limitations

Despite a large sample size of patients with invasively confirmed coronary vessel status and advanced phenotyping of the study cohort using clinical, echocardiographic, and laboratory data, our analysis has some limitations. First, this study was performed as a cross‐sectional analysis. Therefore, association between coronary artery status and incident AF could not be analyzed. Secondly, the study is a single center observational study with patients of European ancestry covering small geographic area in Eastern Germany. Thus, generalizability of the study results to other populations is limited. Thirdly, we could not distinguish AF subtypes, and patients with paroxysmal AF were most likely underdiagnosed. Fourthly, our study cohort included ~66% men, thus the association between AF and CAD or CAS is less powered for women. Also, the impact of CAD origin was analyzed only in patients with single vessel CAD. Therefore, an impact of stenosis oring in double or triple vessel CAD might be different. Further studies with cardiac MRI data analyzing myocardium vitality should provide a link between CAD origin and risk for AF. Furthermore, the drug dosage was unknown in the LIFE‐Heart Study; therefore, the role of high and low dosage drugs was not analyzed. Finally, no follow‐up data were available to analyze association between CAD progression and AF.

## 6 CONCLUSION

In the LIFE‐Heart Study, coronary artery status was associated with risk of AF. Patients with CAS had more often AF compared to patients with unobstructed coronary vessels or CAD. Further studies are required to confirm these findings.

## CONFLICT OF INTEREST

Jelena Kornej received funding from the Marie Sklodowska‐Curie Actions under the European Union's Horizon 2020 research and innovation programme (grant agreement No 838259). Markus Scholz received funding from Pfizer Inc. for a project not related to this research.

## Data Availability

The data that support the findings of this study are available from the corresponding author upon reasonable request.
